# The Relationship Between Macular Cyst Formation and Ischemia in Diabetic Macular Edema

**DOI:** 10.4274/tjo.galenos.2018.19616

**Published:** 2019-09-03

**Authors:** Nuriye Gökçen Yalçın, Şengül Özdek

**Affiliations:** 1 Gazi University Faculty of Medicine, Department of Ophthalmology, Ankara, Turkey

**Keywords:** Diabetic macular edema, diabetic macular ischemia, cystic changes, optical coherence tomography, peripheral ischemia

## Abstract

**Objectives:**

To evaluate the relationship between cyst characteristics and macular and peripheral ischemia in diabetic macular edema (DME).

**Materials and Methods:**

We retrospectively reviewed eyes with DME and included those with clinically significant macular edema as defined by ETDRS (Early Treatment Diabetic Retinopathy Study) and cystoid spaces in optical coherence tomography scans in this study. Central subfield thickness (CSFT), horizontal and vertical diameters of the largest cyst, cyst area, and the remaining retinal thickness outside the cyst were determined. The presence and number of hyperreflective foci in the cyst wall and the internal reflectivity of the cyst were analyzed. Outer retinal damage was graded. Fluorescein angiography was used to determine the areas of macular and peripheral ischemia, which were graded as mild or severe. Correlations between macular and peripheral ischemia and cyst-related measurements and structural changes in the retina were evaluated.

**Results:**

This retrospective study included 250 eyes of 186 patients with DME. Mean CSFT was significantly greater in eyes with macular ischemia (510.4±144.7 μm) compared to eyes without macular ischemia (452.1±114.6 μm) (p=0.001). Horizontal and vertical diameter of the largest cyst increased with the presence and severity of macular ischemia (p=0.045 and p=0.016, respectively). Remaining retinal thickness increased with the presence and severity of peripheral ischemia (p=0.009). There was a statistically significant relationship between the number of the hyperreflective foci in the cyst wall and internal reflectivity of the cyst (p=0.007). Patients with greater CSFT had a 1.04-times higher odds of having macular ischemia and 0.25-times higher odds of having outer retinal damage.

**Conclusion:**

The likelihood of macular ischemia increases with larger cyst diameter, CSFT, and extent of outer retinal damage. Thickness of the noncystic area is increased in the presence of peripheral ischemia.

## Introduction

The most common cause of visual loss in people with diabetes is diabetic macular edema (DME).^1^ There are two different aspects of the diabetic retinopathy (DR) spectrum in terms of retinal vasculature: hyperpermeability (leakage and edema) and hypoperfusion (ischemia).^2^ Ischemia can occur both in the macula and in the peripheral retina. The enlargement of the foveal avascular zone (FAZ) can be described as diabetic macular ischemia (DMI).^3,4^ This definition includes occlusion of retinal capillaries in the macula and obliteration of precapillary arterioles.^4^ DMI is associated with poor visual prognosis.^5,6^

A remarkable association was found between the extent of peripheral nonperfused areas and the degree of DME.^7^ Vascular endothelial growth factor (VEGF), which is the most significant factor in the pathogenesis of DR, is released from ischemic areas.^8^ Neovascularization and increased permeability of the vascular structures occur with the activation of VEGF, which was found in patients with DME.^8,9,10^

Cystoid macular edema (CME) is one of the morphological patterns of DME on optical coherence tomography (OCT).^11^ In the process of cyst formation, fluid accumulates in the intercellular space in the acute phase. Later, in the chronic stage, fluid begins to form in the intracellular space. Thus, this accumulation leads to large cystoid cavities.^12^ Cystoid spaces at the fovea and enlarged FAZ were found to be related to each other.^13^ Besides the common pathogenesis of cyst and ischemia, cyst presence has been linked to decreased retinal sensitivity.^14^ To date, there has been no description in the literature of quantitative and qualitative cyst features associated with retinal ischemia as a part of the degenerative process.

The aim of this study was to investigate the relationship between cyst formation and related OCT features and both macular and peripheral retinal ischemia.

## Materials and Methods

In this retrospective cross-sectional study, medical records of patients who were followed up with the diagnosis of cystic DME at Gazi University Department of Ophthalmology between November 2011 and March 2015 were evaluated for inclusion. The study was approved by the local ethics committee of Gazi University.

Eyes with clinically significant macular edema as defined by the Early Treatment Diabetic Retinopathy Study (ETDRS), cystoid spaces in OCT scans, and high-quality fluorescein angiography (FA) and spectral domain images were included in the study. Eyes with macular edema due to other causes such as uveitis; retinal vein occlusion; concurrent macular degeneration; macular hole; visually significant cataract or any other pathology causing visual loss such as amblyopia, corneal opacity, significant vitreous hemorrhage, and optic atrophy were excluded from the study. Eyes that had undergone cataract surgery within the last 6 months were also excluded from the study to exclude Irvine-Gass syndrome.

The demographic features of the patients (age, sex, duration of diabetes) and stage and duration of DR were recorded. The records of patients were reviewed for best corrected visual acuity (BCVA), DR findings in fundus examination, OCT and FA evaluation. BCVA was converted to LogMAR for statistical analysis. All OCT scans and FA investigations were performed with Heidelberg Spectralis OCT (Spectralis; Heidelberg Engineering, Heidelberg, Germany). All images were obtained by the same experienced technician. In OCT, 25-line raster scans were obtained for each eye after pupil dilation and the average thickness in the central 1000-µm diameter circle of the ETDRS grid was accepted as central subfield thickness (CSFT).

Cystic space was defined as round or oval-shaped low reflective intraretinal spaces separated by hyperreflective septa.^11^ In the analyses of cysts in OCT, we used the largest cystoid space in the area within 1000 µm of the foveal center as representative of degenerative status. The horizontal and vertical diameters of the largest cyst, the area of the cyst (product of the diameters), and the remaining retinal thickness outside the cyst were determined in the quantitative analyses part of the study. Remaining retinal thickness outside the cyst was calculated by subtracting the vertical diameter of the cyst from the CSFT. All measurements were performed by the same investigator (N.G.Y.) with a manual caliper.

In the qualitative examination of the cyst, the presence and the number of hyperreflective foci in the cyst wall and the internal reflectivity of the cyst were analyzed. Hyperreflective foci were defined as the hyperreflective dots less than 30 µm in thickness and having the same reflectivity of clustered hard exudates, as described by Bolz et al.^15^ The internal reflectivity of the cysts was classified as isoreflective when similar to the retinal layers, hyporeflective when similar to the vitreous, or heterogeneous (as described in an earlier study).^16^ Accompanying outer retinal damage in the ellipsoid zone was also determined and any loss of continuity of either the external limiting membrane (ELM) or inner segment/outer segment (IS/OS) band in the central 0.1 mm of the fovea was noted as outer retinal damage.^17^

**Figure 1:**
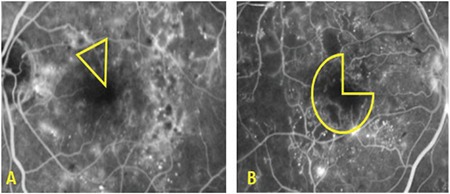
Macular ischemia: A) Mild ischemia, disruption of less than half of the foveal avascular zone (FAZ) outline, B) Severe ischemia, disruption of more than half of the FAZ outline

**Figure 2:**
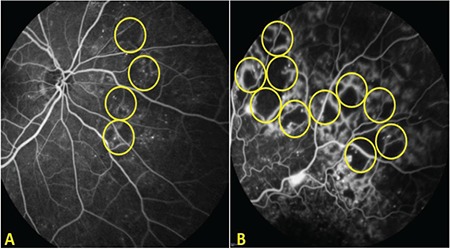
Peripheral ischemia: A) Mild ischemia, covers less than a 5-disc diameter area, B) Severe ischemia, covers more than a 5-disc diameter area

Macular and peripheral ischemia were assessed from FA images. Macular ischemia was defined as an enlarged FAZ (≥1000 µm) or presence of capillary nonperfusion within one disc diameter (DD) from the foveal center.^18^ The severity of macular ischemia was graded according to disruption of the FAZ outline. If the ischemic area affected less than half of the FAZ outline, it was evaluated as mild; further disruption was graded as severe (Figure 1).^19^ Peripheral ischemia was defined as hypofluorescent areas corresponding to retinal nonperfusion/capillary drop-out or intraretinal microvascular anomaly in at least a 1-DD area.^7^ It was graded as mild when the peripheral ischemia covered less than a 5-DD area and as severe when it was more than a 5-DD area when evaluated on images taken in all gaze directions (Figure 2). This cut-off level was chosen because it was shown that the risk of neovascularization emerged over this value.^20^

### Statistical Analysis

Data obtained from the study were recorded using Excel for Windows (version 2010, Microsoft, Redmond, WA) and statistical analyses were performed using the Statistical Package for the Social Sciences for Windows (version 15.0, SPSS, Chicago, IL). The statistical level of significance was set to p>0.05. Kolmogorov-Smirnov test, histograms, and P-P plots were used to test continuous variables for conformity to normal distribution. One-way ANOVA and LSD test for post-hoc analyses were used for the comparison of three or more groups if the variables were normally distributed. The Kruskal-Wallis test was used for the comparison of three or more groups if the variables were not normally distributed. Mann-Whitney U test with Bonferroni correction was used for post-hoc analysis if the result revealed a significant difference. Pearson chi-square or Yate’s corrected chi-square tests were used for categorical variables. Binary logistic regression analyses were done.

## Results

A total of 250 eyes of 186 patients met the inclusion criteria. There were 64 patients (34.4%) with bilateral involvement and 122 patients (65.6%) with unilateral DME. One hundred ninety-four eyes (77.6%) had received prior intravitreal injection and/or laser therapy. Other demographic features of the cases are shown in Table 1. The mean BCVA of the patients was 0.5±0.38 (0-1.6) LogMAR.

Macular ischemia was present in 110 eyes (44%). Seventy-two eyes (28.8%) had mild macular ischemia and 38 eyes (15.2%) had severe macular ischemia. The relationship between DR stage and ischemia is shown in Table 2. Mean BCVA was 0.36±0.28 LogMAR in eyes with normal macular perfusion and 0.68 ± 0.42 LogMAR in eyes with macular ischemia (p=0.001). The mean CSFT was 510.35±144.68 µm in the eyes with macular ischemia, which was significantly higher than that of the eyes with normal macular perfusion (452.11±114.61 µm) (p=0.001). Outer retinal damage was also more prevalent in eyes with macular ischemia and prevalence increased with ischemia severity (Table 3). Severity of macular ischemia and related OCT features are given in Table 3.

Peripheral ischemia was present in 192 eyes (76.8%). Half of these eyes (96 eyes, 38.4%) had mild peripheral ischemia and the other half (96 eyes, 38.4%) had severe peripheral ischemia. The mean BCVA was 0.43±0.35 LogMAR in eyes without peripheral ischemia and 0.52±0.39 LogMAR in eyes with peripheral ischemia (p=0.076). The mean CSFT was 483.91±138.7 µm in eyes with peripheral ischemia and 457.31±103.5 µm in eyes without peripheral ischemia (p=0.36). Severity of peripheral ischemia and related OCT features are given in Table 4.

Hyperreflective foci in the cyst wall were detected in 170 eyes (68%). Most of these hyperreflective foci (155 eyes, 91%) were in the outer retinal layers. The median number of the foci was 1 (0-14) in the cyst wall and 1 (0-8) in outer retinal layers. The number of the hyperreflective foci did not change significantly with the severity of macular or peripheral ischemia (p>0.05).

Internal reflectivity of the cyst did not differ significantly between eyes with and without ischemia (p>0.05). Eighty-two eyes (33%) had hyporeflective cysts and 60 (24%) had isoreflective cysts. Heterogeneous internal reflectivity was observed in 108 eyes (43%). The number of hyperreflective foci in the cyst wall was significantly higher in the isoreflective internal reflectivity group than the others (p=0.007) (Table 5).

In binary logistic regression analyses, only CSFT and outer retinal damage status were statistically significant for macular ischemia. No significant risk factor for peripheral ischemia was identified in binary logistic regression analyses (Table 6).

## Discussion

Pericyte loss and autoregulatory dysfunction play an important role in the pathophysiology of DR. Thus, weakening and destruction of retinal vessels occur.^6^ Hyperpermeability and ischemia are different components of DME and also the main outcomes of the distorted vascular network. In an earlier study, the presence of a cyst was associated with decreased retinal sensitivity independent of IS/OS damage and increased retinal thickness.^14^ In this study, we focused on the impact of diabetic cystic changes on the ischemic process.

Enlargement of the FAZ and perifoveal intercapillary area and disruption of macular circulation have already been demonstrated in conjunction with the progression of DR and visual disturbance.^5,13,21,22,23^ In the present study, severe peripheral retinal ischemia was more prevalent in eyes with proliferative DR, as expected. However, no statistically significant difference was observed in the prevalence of macular ischemia between the groups according to the severity of DR or peripheral retinal ischemia.

Macular ischemia was found to be more prevalent in eyes with larger macular cysts and CSFT. Larger FAZ areas have been observed in the superficial, deep, and summated capillary plexus in diabetic patients in several studies using OCT angiography (OCTA), which is one of the current retinal imaging methods and allows construction of microvascular flow maps.^24,25,26,27^ Also, similar to our study, disorganization and loss of retinal capillaries was observed more precisely in the deep plexus with more severe DME in OCTA images.^28^ Although enlargement of the cyst in both planes seems to be associated with macular ischemia, vertical enlargement and retinal thickness had a greater impact on the ischemic process. However, we believe that the horizontal enlargement of the cyst is associated with degeneration of Muller cells. In contrast to this hypothesis, a study that classified diabetic CME based on the ratio of vertical size of the largest cyst to the maximum macular thickness showed that disruption of the cystic septa occurred in the most advanced stage.^29^ In a previously published study, vascular hyperpermeability and ischemia were shown to cause necrosis and apoptosis in the neuroglia cells, resulting in large cystoid cavities. A vicious circle ensues, with the enlargement of the cystoid spaces causing enlarged FAZ and increased foveal ischemia.^13^ In the same study, sponge-like retinal thickening and larger FAZ were more common in CME cases than in serous foveal detachment.

We hypothesized that the disruption of retinal structures and the ischemic process as a part of the degeneration occur together in the chronic stage of DME. The damaging effect of cyst formation on ganglion and bipolar cells has been suggested in previously published studies.^12,30,31^ The presence of macular cystoid spaces was found to be predictive of visual deterioration, with larger cystoid spaces being more disruptive than small ones.^32^ In light of this information and the findings in this study, larger cysts may have a damaging effect that triggers or exacerbates the ischemic state. Regression analyses more clearly demonstrated the association between CSFT and outer retinal disruption and macular ischemia. Patients with more severe DME had a 1.04-fold greater chance of having macular ischemia, and those with outer retinal damage had a 0.25-fold greater chance of having macular ischemia. Furthermore, outer retinal damage was observed more frequently in severe macular ischemia.

We have shown that as a result of the degenerative process, BCVA decreased gradually with increasing severity of ischemia. Similarly, Koleva-Georgieva and Sivkova^33^ demonstrated a negative correlation between BCVA and cystoid DME groups (classified according to the horizontal diameter of cystoid spaces as mild <300 µm, intermediate 300-600 µm; and severe >600 µm). Both macular ischemia and cyst size affect visual acuity.

In our study, qualitative parameters such as the presence and number of hyperreflective foci in the cyst wall and internal reflectivity of the cyst were not associated with macular or peripheral ischemia.

Hyperreflective foci defined by Bolz et al.^15^ were suggested to be subclinical characteristics of lipoprotein extravasation and an early manifestation of DME. Hyperreflective foci that can be found scattered throughout all retinal layers have also been detected in the cystoid space. They were correlated with modest fluorescein pooling and heterogeneous reflectivity.^34^ In the current study, we studied hyperreflective foci in the cyst wall and hypothesized that they may be associated with the early period of degenerative process and ischemia. Although not associated with ischemia, we found a significant relationship between the number of hyperreflective foci in the cyst wall and the internal reflectivity of the cyst. It was hypothesized that internal reflectivity of cysts was related with degeneration; in the early period, the cyst is usually isoreflective, then becomes heterogeneous due to the debris accumulation resulting from degeneration, and finally, the degenerated cyst becomes hyporeflective in the chronic stage. Our findings supported this hypothesis in that there were more hyperreflective foci in the isoreflective cysts.

The relationship between macular edema and peripheral ischemia was inconclusive. We failed to show any relationship between the presence and severity of the peripheral ischemia and CSFT. Similarly, the severity of DME was not found to be correlated with the global nonperfusion area in the DAVE study.^35^ In contrast to these findings, Wessel et al.^7^ claimed that the risk of having DME increases 3.75 times in the presence of ischemia. However, in the same study there was no relationship between degree of ischemia and DME. Similar to this study, peripheral ischemia was shown to be associated with greater degree of DME in a study using ultra-wide-field angiography (UWFA).^36^ In the current study, there was a statistically significant relationship between the noncystoid retinal tissue and peripheral ischemia. Noncystoid retinal thickness increased with the presence and severity of ischemia in the peripheral retina. This may be explained by a diffuse thickening of the macula outside the cyst due to increased VEGF load in the presence of peripheral ischemia.

### Study Limitation

The retrospective design is the main limitation of our study. Other limitations include unavailability of current retinal imaging methods that can be used for ischemia such as OCTA and UWFA in our clinic and the lack of image analysis software for the measurement of nonperfusion areas and calculation of ischemic index.

## Conclusion

In conclusion, the possibility of macular ischemia increases when the diameter of the cyst increases. The main factors increasing the probability of ischemia were increased CSFT and the presence of outer retinal damage. In cystoid DME, greater CSFT is associated with larger cyst, more outer retinal damage, and higher likelihood of macular ischemia findings in FA. In addition, the presence of peripheral ischemia seems to increase retinal thickening in the noncystic retinal area.

## Figures and Tables

**Table 1:**
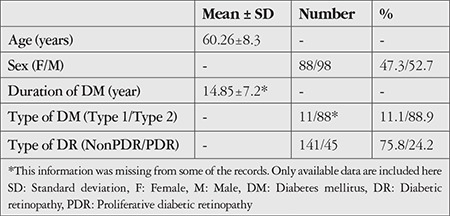
The demographic and clinical features of the patients

**Table 2:**
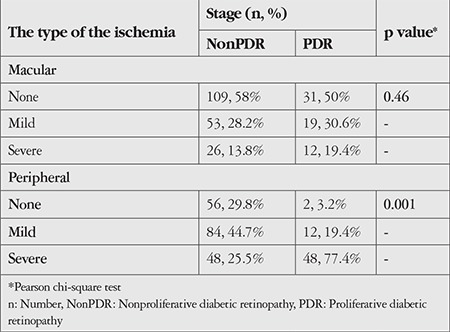
Macular and peripheral ischemia according to the stages of diabetic retinopathy

**Table 3:**
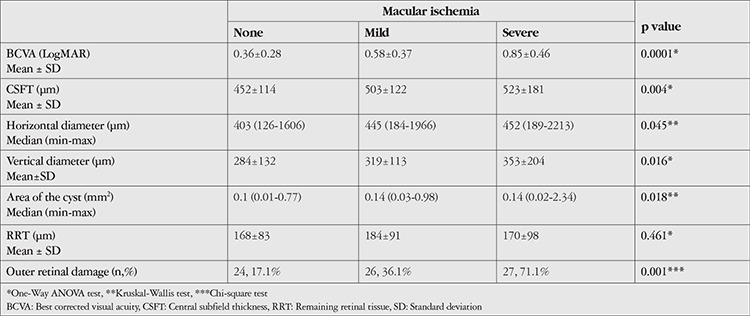
Best corrected visual acuity and optical coherence tomography findings according to presence of macular ischemia

**Table 4:**
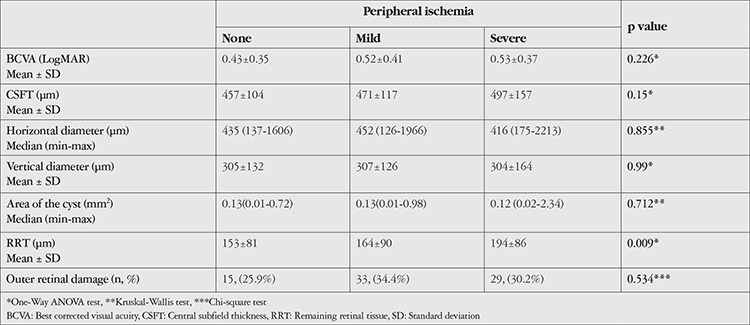
Best corrected visual acuity and optical coherence tomography findings according to presence of peripheral ischemia

**Table 5:**
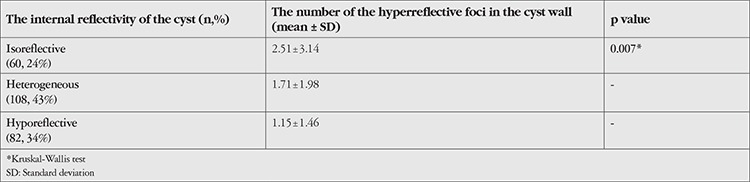
The relationship between the internal reflectivity of the cyst and the number of the hyperreflective foci in the cyst wall

**Table 6:**
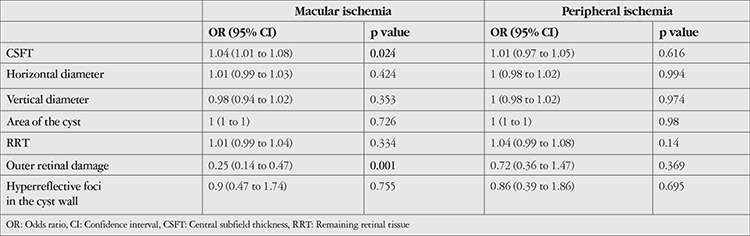
The odds of macular and peripheral ischemia in patients with diabetic cystic changes
